# Seroprevalence of leptospirosis among blood donors in an endemic area

**DOI:** 10.1038/s41598-023-39461-3

**Published:** 2023-07-31

**Authors:** Umaporn Limothai, Sasipha Tachaboon, Janejira Dinhuzen, Jasleen Singh, Sirawit Jirawannaporn, Akarathep Leewongworasingh, Matsaya Thongpin, Suppalak Brameld, Phandee Watanaboonyongcharoen, Visith Sitprija, Terapong Tantawichien, Usa Thisyakorn, Nattachai Srisawat

**Affiliations:** 1 Excellence Center for Critical Care Nephrology, King Chulalongkorn Memorial Hospital, Bangkok, Thailand; 2 Center of Excellence in Critical Care Nephrology, Faculty of Medicine, Chulalongkorn University, Bangkok, Thailand; 3 Tropical Medicine Cluster, Chulalongkorn University, Bangkok, Thailand; 4 School of Global Health, Faculty of Medicine, Chulalongkorn University, Bangkok, Thailand; 5 Thailand Public Health Research Fellowship, Health Education England, London, UK; 6 National Institute of Health of Thailand, Nonthaburi, Thailand; 7 Department of Laboratory Medicine, Faculty of Medicine, Chulalongkorn University and Transfusion Medicine Unit, King Chulalongkorn Memorial Hospital, Bangkok, Thailand; 8 Thai Red Cross, Queen Saovabha Memorial Institute, Bangkok, Thailand; 9 Division of Infectious Diseases, Department of Medicine, Faculty of Medicine, Chulalongkorn University, Bangkok, Thailand; 10 Division of Nephrology, Department of Medicine, Faculty of Medicine, King Chulalongkorn Memorial Hospital, Bangkok, Thailand; 11 Center for Critical Care Nephrology, The CRISMA Center, Department of Critical Care Medicine, School of Medicine, University of Pittsburgh, Pittsburgh, PA USA; 12 Academy of Science, Royal Society of Thailand, Bangkok, Thailand; 13 Excellence Center for Critical Care Nephrology, Thai Red Cross Society, King Chulalongkorn Memorial Hospital, 1873, Rama 4 Rd., Lumphini, Pathumwan, Bangkok, 10330 Thailand

**Keywords:** Infectious-disease epidemiology, Health policy, Epidemiology, Population screening

## Abstract

Thailand is known to be endemic for leptospirosis. This bacterium may pose a potential risk to transfusion safety. This study was a cross-sectional study examining the seroprevalence of leptospirosis among Thai blood donors. A total of 1053 serum specimens collected from blood donors residing in 5 regions of Thailand during March to September 2020 were included in this study. All samples were tested for the presence of antibodies to 22 leptospiral serovars using the microscopic agglutination test (MAT) and anti-*Leptospira* IgG antibodies using commercially available enzyme immunoassay. We found no evidence of recent exposure to *Leptospira* spp. in sera of healthy Thai blood donors by MAT, including those in higher-risk areas. However, in this same group, we did find small numbers of past exposure (1.7%) to *Leptospira* spp*.* by IgG ELISA. According to the findings of this study, there is currently no evidence for implementing new blood banking procedures to identify possible carriers in Thailand, however these should be continually monitored and revised according to the infectious disease burden in each country. It should be noted that there was a difference in the occupation rate between the general population reported in Thailand and blood donors in this study; it may not reflect the actual situation in the country.

## Introduction

Leptospirosis is a neglected tropical disease and one of the most common bacterial zoonoses worldwide, causing an estimated 1.03 million cases and 58,900 deaths annually^1^. The disease is endemic in many tropical countries and often has a seasonal distribution, increasing with heavy rain and higher temperatures^2^.

Leptospirosis is a major public health problem in Thailand, with an average annual incidence rate of 3.19 cases per 100,000 population and a case fatality rate of 0.04 cases per 100,000 population from 2020 to 2022^3^. Most leptospirosis-confirmed cases occur in the northeast and south regions of the country^3^, with the highest incidence during the rainy season. Most reported cases are in agricultural workers such as rice cultivators, who are likely to be exposed to contaminated environments during their daily activities^4–6^. Various mammals such as rodents, livestock, and domestic pet act as reservoir hosts, and infections are acquired through infected urine or a contaminated environmental source, typically floodwater^7,8^.

The causative agents of leptospirosis are spirochetes of the genus *Leptospira* of which there are more than 60 species have been described for the entire genus *Leptospira*, including 17 pathogenic species^9^. Clinical infection presents in a wide variety of manifestations ranging from a mild influenza like illness to serious illness including Weil’s syndrome (characterized by jaundice, renal failure and myocarditis), meningitis and pulmonary haemorrhage^10^. The infection has an incubation period of 5–14 days with a range of 2–30 days. Symptoms mimic many other diseases such as dengue, influenza and viral haemorrhagic diseases^8^.

Many infectious disease agents pose a risk to transfusion safety^11^. The risk of transfusion-transmitted infections is lower than previously due to strict regulations on the use of blood products, including risk-based assessments for donors and screening of blood products^12,13^. However, the transmission of infectious diseases through blood products is still a major concern worldwide, and as a high-risk area, it is possible that leptospirosis could pose a risk to transfusion safety in Thailand. There is evidence that transmission of *Leptospira* by blood transfusion is possible^14,15^. One case has been reported in India, indicating that blood transfusion from an asymptomatic carrier donor can result in the transmission of leptospirosis in the recipient^16^.

This is a cross sectional, descriptive study of leptospirosis seroprevalence in Thailand. It has relevance since leptospirosis is an important infectious disease in tropical regions such as Asia, and clinical diagnosis may be missed in areas where dengue or influenza are also endemic. Blood transfusion poses an important risk for infectious disease transmission as has been seen with HIV, malaria and hepatitis in regions where they are endemic, and it is justified to screen for common diseases in blood banks given the risk of transmission. We therefore examined leptospirosis seroprevalence rates among a large cohort of healthy Thai blood donors. This research adds important evidence to inform decision making regarding transfusion transmitted infections.

## Results

### Demographic data

A total of 1053 serum specimens from 5 regions of Thailand were included in this study. The demographic characteristics of the studied population are presented in Table 1. Overall, 50% of the samples were from male donors, and the median age was 35 years (range, 18–66 years). They were equally collected from 5 provinces representing five regions of Thailand. Most donors were office workers (35.9%) with no underlying disease.

**Table 1:** Demographic characteristics of *Leptospira* IgG ELISA-positive donors in Thailand.

Characteristic	All donors, N (%)	Prevalence of anti-*Leptospira* IgG positive, N (%)
Gender		
Female	526 (50.0)	6 (1.1)
Male	527 (50.0)	12 (2.3)
Missing data	0 (0.0)	0 (0.0)
BMI		
Median (IQR)	24.1 (22.0–26.9)	24.1 (21.2, 28.3)
Underweight (< 18.5)	22 (2.2)	0 (0.0)
Normal (18.5–22.9)	358 (35.1)	6 (1.7)
Overweight (23–24.9)	218 (21.4)	4 (1.8)
Pre-obese (25–29.9)	304 (29.8)	4 (1.3)
Obese (> = 30)	117 (11.5)	3 (2.6)
Missing data	34 (3.2)	1 (2.9)
Age		
Median (IQR)	35.0 (28.0, 44.0)	34.5 (30.0, 40.3)
18–20	36 (3.4)	0 (0.0)
21–30	316 (30.0)	6 (1.9)
31–40	334 (31.7)	8 (2.4)
41–50	252 (24.0)	4 (1.6)
> 50	114 (10.8)	0 (0.0)
Missing data	1 (0.1)	0 (0.0)
Living area		
Bangkok	218 (20.7)	4 (1.8)
Khon Kaen	201 (19.1)	1 (0.5)
Chonburi	233 (22.1)	4 (1.7)
Chiang Mai	201 (19.1)	4 (2.0)
Phuket	200 (19.0)	5 (2.5)
Missing data	0 (0)	
Occupation		
Agricultural	3 (0.3)	0 (0.0)
Arts, design, entertainment, sports, and media	3 (0.3)	0 (0.0)
Government	111 (10.8)	1 (0.9)
Office workers	370 (35.9)	8 (2.2)
Education	19 (1.8)	0 (0.0)
Military	17 (1.7)	0 (0.0)
Merchant	71 (6.9)	1 (1.4)
Health care workers	23 (2.2)	2 (8.7)
Student	72 (7.0)	0 (0.0)
Commercial services staff	26 (2.5)	1 (3.8)
Legal	3 (0.3)	0 (0.0)
Food preparation and serving related workers	5 (0.5)	0 (0.0)
Household employee	37 (3.6)	1 (2.7)
Factory worker	2 (0.2)	0 (0.0)
Others	243 (23.6)	2 (0.8)
Monkhood	6 (0.6)	1 (16.7)
Unemployed	12 (1.2)	0 (0.0)
Transportation and material moving	7 (0.7)	1 (14.3)
Missing data	23 (2.2)	0 (0.0)
Underlying disease	75 (7.1)	1 (1.3)
Allergy	21 (2.0)	1 (4.8)
Gastritis	5 (0.5)	0 (0.0)
Dyslipidemia	8 (0.8)	0 (0.0)
Diabetes mellitus	4 (0.4)	0 (0.0)
Gout	4 (0.4)	0 (0.0)
Hypertension	24 (2.3)	0 (0.0)
Migraine	5 (0.5)	0 (0.0)
Peripheral neuropathy	1 (0.1)	0 (0.0)
Polycystic kidney disease	1 (0.1)	0 (0.0)
Sinuses	1 (0.1)	0 (0.0)
Acute stress disorder	1 (0.1)	0 (0.0)
Meniere’s disease	1 (0.1)	0 (0.0)
Epilepsy	1 (0.1)	0 (0.0)
Missing data	0 (0)	0 (0.0)

### Seroprevalence of leptospirosis among blood donors

None of the 1053 serum samples had antibody titers suggestive of a recent infection by MAT. However, 18 donors (1.7%) were positive for anti-*Leptospira* IgG antibodies by ELISA. The median age of donors with *Leptospira* IgG ELISA-positive was 34 years (range 27–49 years), and 66.7% were male.

There were no statistically significant differences in the gender (*p* = 0.155), age (*p* = 0.458), living area (*p* = 0.624), and occupation (*p* = 0.074) of the individuals between Leptospira IgG ELISA-positive and Leptospira IgG ELISA-negative. The detailed information of donors who were Leptospira IgG ELISA reactive was shown in Table 2.

**Table 2:** The detailed information of donors who were *Leptospira* IgG ELISA reactive.

No	Code	Collection site	ABO blood group	Rh type	Age	Gender	BMI	Occupation	Underlying disease	History of blood transfusions
1	CUSV 4	Bangkok	Not available	Not available	36	Female	23.19	Office worker	No	No
2	CUSV 121	Bangkok	Not available	Not available	48	Female	27.53	Nurse	No	Yes
3	CUSV 123	Bangkok	Not available	Not available	28	Male	40.40	Taxi driver	No	No
4	CUSV 188	Bangkok	Not available	Not available	37	Male	24.54	Monkhood	No	No
5	CBSV 60	Chonburi	A	Positive	30	Male	NA	Office worker	Allergy	No
6	CBSV 121	Chonburi	O	Positive	38	Male	20.62	Employee	No	No
7	CBSV 122	Chonburi	B	Positive	33	Male	29.07	Office worker	No	No
8	CBSV 218	Chonburi	AB	Positive	30	Female	21.37	Office worker	No	No
9	KKSV 113	Khon Kaen	B	Positive	44	Male	24.09	Nurse	No	No
10	CMSV 107	Chiang Mai	O	Positive	49	Female	18.59	Housekeeper	No	No
11	CMSV 110	Chiang Mai	B	Positive	33	Male	23.66	Merchant	No	No
12	CMSV 111	Chiang Mai	O	Positive	27	Male	32.60	Employee	No	No
13	CMSV 153	Chiang Mai	A	Positive	40	Male	35.86	Government	No	No
14	PUSV 37	Phuket	O	Positive	28	Male	25.01	Office worker	No	No
15	PUSV 40	Phuket	O	Positive	32	Female	20.06	Hotel receptionist	No	No
16	PUSV 92	Phuket	O	Positive	37	Female	20.96	Office worker	No	No
17	PUSV 138	Phuket	O	Positive	41	Male	26.12	Office worker	No	No
18	PUSV 160	Phuket	B	Positive	30	Male	22.79	Office worker	No	No

### Factors associated with Leptospira IgG-positive ELISA

Logistic regression analysis was used to assess the factor associated with the presence of anti-*Leptospira* IgG antibodies. However, the result indicated that there was no significant effect of age, gender, BMI, or region on the rates of *Leptospira* IgG ELISA-positive (p < 0.05).

## Discussion

Thailand is known to be endemic for leptospirosis. This bacterium may pose a potential risk to transfusion safety, especially in acute asymptomatic cases which may favour transfusion-related transmission. In this study, we found no evidence of recent exposure to *Leptospira* spp. in sera of healthy Thai blood donors by MAT, including those in higher-risk areas. However, in this same group, we did find small numbers of past exposure (1.7%) to *Leptospira* spp. by IgG ELISA.

There are possible reasons for the inconsistent result between MAT and ELISA from our findings. MAT is one of the gold standard methods for diagnosing leptospirosis which detects serovar-specific antibodies^17^, however there are limitations in using this method. Previous studies (including one in Thailand) have demonstrated low sensitivity of MAT as compared to MAT with culture^18^. Indeed, research has demonstrated that regional *Leptospira* serovar-specific IgG ELISA is superior to MAT in diagnosing leptospirosis^19^. There are several potential reasons for the low performance of MAT, including that it may take several weeks after infection for specific antibodies against the bacteria to reach detectable levels^20^. In addition, MAT uses a live panel of *Leptospira* representing the main serovars and for proper performance of the test, an optimized panel of antigens must be selected^21^. The use of MAT therefore requires an accurate knowledge of local circulating serovars with regular surveillance to maintain a complete panel of relevant antigens^22^. It is therefore possible that in our study the panel of antigens used in the MAT did not cover locally circulating serovars in Thailand. In addition, *Leptospira* contamination of donated blood occurs when the donor is in the early stages of infection. At this time, the donor has not yet produced sufficient antibodies and may not be detected using MAT. Regarding IgG ELISA, we used a commercially available kit that uses a native membrane extract from *Leptospira biflexa* with genus-specific antigens. Therefore, the kit can detect IgG antibodies directed against all *Leptospira* spp including pathogenic *Leptospira*. For this reason, the IgG ELISA detection rate may be higher than the MAT. However, information regarding the exact protein of L. biflexa that the kit used as an antigen was not provided by the company.

Data from the Thailand Ministry of Public Health from 2003 to 2012 indicated that the incidence rates of leptospirosis were highest in the northeastern region^23^. However, there has been a recent shift with a higher incidence of leptospirosis reported in the southern region since 2020^3^. There are significant environmental and cultural differences between the northeastern and southern regions of Thailand including in people’s lifestyles, housing, and ecology^24^. Northeast Thailand is a high-flat plain and relatively low humidity. Households frequently share various water sources for agricultural usage. Most of the study population in this region worked in fields frequently exposed to animals and environmental bodies of water, like rice paddy fields. Southern Thailand is high humidity and heavy rains, mainly covered in tropical rainforests. Instead of working in livestock farming or other jobs involving animals, most people work on rubber or palm plantations^24^. However, *Leptospira* IgG detection rates in our cohort of blood donors did not vary by area.

Seroprevalence data of leptospirosis in the Thai general population are limited. The first nationwide leptospirosis seroprevalence study (using agglutination tests) was conducted in 1966 on nonfebrile adult patients in hospitals across Thailand^25^. This study reported positive agglutination reactions for various serotypes of leptospirosis in 22–35% of the 3746 people examined^25^. A second study (using *Leptospira* IgG ELISA) was conducted on repository serum specimens obtained from young Thai men entering the Royal Thai Army without suspicious symptoms during 2007–2008 and found an overall seroprevalence rate of 28%^26^. Recently, Chadsuthi et al. used MAT to analyze Leptospira seroprevalence in 1990 human serum samples under suspicion of leptospirosis collected from 5 regions of Thailand between 2010 and 2015, among these 23.7% were found seropositive^27^.

We used a health blood donor population as a proxy for the general population, and there is a risk that our included population may not be the population at high risk of leptospirosis. Blood donor based serosurveillance has long been used as a powerful tool and cost-effective strategy providing insights on past emerging infectious threats such as West Nile, dengue, chikungunya, Zika and more recently COVID-19^28–34^. However, care must always be taken when extrapolating data from donor seroprevalence studies to the general population, as blood donor demographics will be different to those of the general population.

In our study we looked at a cohort of blood donors, a population that represents a potential source of transmission of leptospirosis through blood transfusion. This risk is low, with only one such case previously described in India^16^, however the risk of blood borne transmission from infectious diseases such as leptospirosis that can present asymptomatically (meaning that carrier status is difficult to detect) necessities strict enforcement of blood donors screening practices. This is particularly relevant in areas where adequate screening of blood products may not be performed.

Fortunately, Thailand has a strong haemovigiliance system in place adhering strictly to standard WHO guidelines including implementing a strategic plan, relevant legislation and regular inspections of facilities. The Thailand Blood Centre works with the Thai Red Cross Society in providing transfusion services across the country through a National Blood Center, 12 Regional Blood Centers, 166 blood service branches and a plasma fractionation center^35^. In 2010, the Thailand National Blood Centre in cooperation with the Ministry of Public Health created the National Blood Policy which aimed to provide safe blood for patients in accordance with the principles of the WHO; namely by recruiting blood donations from a low-risk population, screening blood donors, standardised testing of all units of blood, and conducting a compatibility test for ensuring safe transfusion^36^. For the safety of donors and recipients of blood transfusion, all donors need to answer a questionnaire which includes general health and conditions that might increase infection risk^36^. The standard infections screened for in Thailand include HIV 1/2 and HIV p24 antigen, HBsAg, anti-HCV ELISA, Rapid Plasma Reagin (RPR) or Treponema Pallidum Hemagglutination Asssay (TPHA) and syphilis antibodies^36^. Additionally, NAT is used for HIV, HBV, and HCV in the negative serology screening test unit as a sequential test. *Leptospira* is not considered in screening, as per global haemovigilance standards. Our findings of low prevalence of serum leptospirosis among blood donors from highly endemic areas therefore provide support for current Thai blood donor screening practices.

In this study, we could not identify factors associated with past exposure to *Leptospira* spp. which may be related to the small number of IgG positive cases. An estimated leptospirosis seroprevalence of 28% based on a previous study in young men entering the Thai Army was used for our sample size calculations^26^.

There are several strengths to our study. Whilst there has been similar research conducted in other countries, this is the first attempt at quantifying the seroprevalence of leptospirosis among a healthy blood donor population in Thailand, an endemic area for leptospirosis. Our data included a large sample size of 1053 serum specimens which is one of the largest cohorts of blood donors tested for seroprevalence of leptospirosis that we have found in the published literature^14,37–39^ as illustrated in Table 3.

**Table 3:** Studies for estimation of *Leptospira* seroprevalence in blood donors.

Study place	Study year	Sample size	Methodology	Results
Brazil^14^	1995	2368	IgM-ELISA, MAT, Macroscopic agglutination test, confirmed by immunoblotting	IgM-ELISA = 42 (1.8%), 24 (1.01%) were confirmed by immunoblotting), MAT (titer ≥ 1:50) = 20, Macroscopic agglutination test = 24
Australia^37^	2009 and 2011	485	MAT	MAT (titer ≥ 1:50) = 1.44%
Peru^39^	2014	42 donors with inclusion criteria were tested	PCR	PCR = 19%
India^40^	2019	100	DMF, MAT, PCR	DMF = 31%, MAT (Cut-off titer was not defined in this paper) = 40%, PCR = 0%
This study	2020	1053	MAT and IgG ELISA	MAT (titer ≥ 1:50) = 0%, IgG ELISA = 1.7%

*MAT* microscopic agglutination test, *ELISA* enzyme-linked immunosorbent assay, *PCR* polymerase chain reaction, *DMF* dark-field microscope, *IgM* Immunoglobulin M, *IgG* Immunoglobulin G.

Our study does have several limitations. Firstly, we were not able to collect data on exposure history such as direct contact with body fluids or organs of infected animals, as well as routes of infection through additional sources such as indirectly through contaminated soil or water. Secondly, the present study did not perform a qPCR test. Therefore, we might miss some acute leptospirosis infection cases and further studies are needed on using a qPCR-based molecular method as a complementary test to MAT and ELISA. Finally, leptospiral infections are typically high-risk among outdoor workers, especially agricultural workers; however, in this study, we focused on the seroprevalence of leptospirosis in a blood donor population to assess the potential risk to transfusion safety. We used a random sampling method that randomly selects participants from the blood donor population even if they may not be a high-risk group. Therefore, the results should be interpreted with a degree of caution. Regarding occupational backgrounds, one of the largest studies which included 30,115 samples obtained from the National Blood Center in Bangkok and two Regional Blood Centers in Lop Buri province and Chon Buri province indicated that most of the donors were private sector workers (63%) followed by government workers (13%) and students (12%)^41^. Agricultural workers were classified as ‘other’, accounted for less than 12% of included donors^41^. However, it should be noted that the occupation trend in blood donors may be different from the general Thai population^41^.

Overall, our findings provide support for the appropriateness and effectiveness of current relevant Thailand donor selection policies and suggest that, even in areas with a relatively high incidence of leptospirosis, this bacterium does not currently seem to be a primary concern for blood services.

## Conclusions

Leptospirosis remains a highly endemic infectious disease in Thailand. Current blood donor screening programs do not include detection of *Leptospira spp*. The seroprevalence of leptospirosis among a cohort of healthy Thai blood donors is low with no donors demonstrating acute infection with leptospirosis, and small numbers (1.7%) demonstrating evidence of past exposure. According to the findings of this study, there is currently no evidence for implementing new blood banking procedures to identify possible carriers in Thailand, however these should be continually monitored and revised according to the infectious disease burden in each country. It should be noted that there was a difference in the occupation rate between the general population reported in Thailand and blood donors in this study; it may not reflect the actual situation in the country.

## Methods

### Study design and population

The study was a cross-sectional study examining the seroprevalence of leptospirosis among Thai blood donors. An initial sample size of 861 for the study was determined, based on an estimated leptospirosis seroprevalence of 28%^26^ at a confidence level of 95% and precision of 3%. A total of 1053 samples were tested, including indeterminate and invalid results.

**Figure 1:**
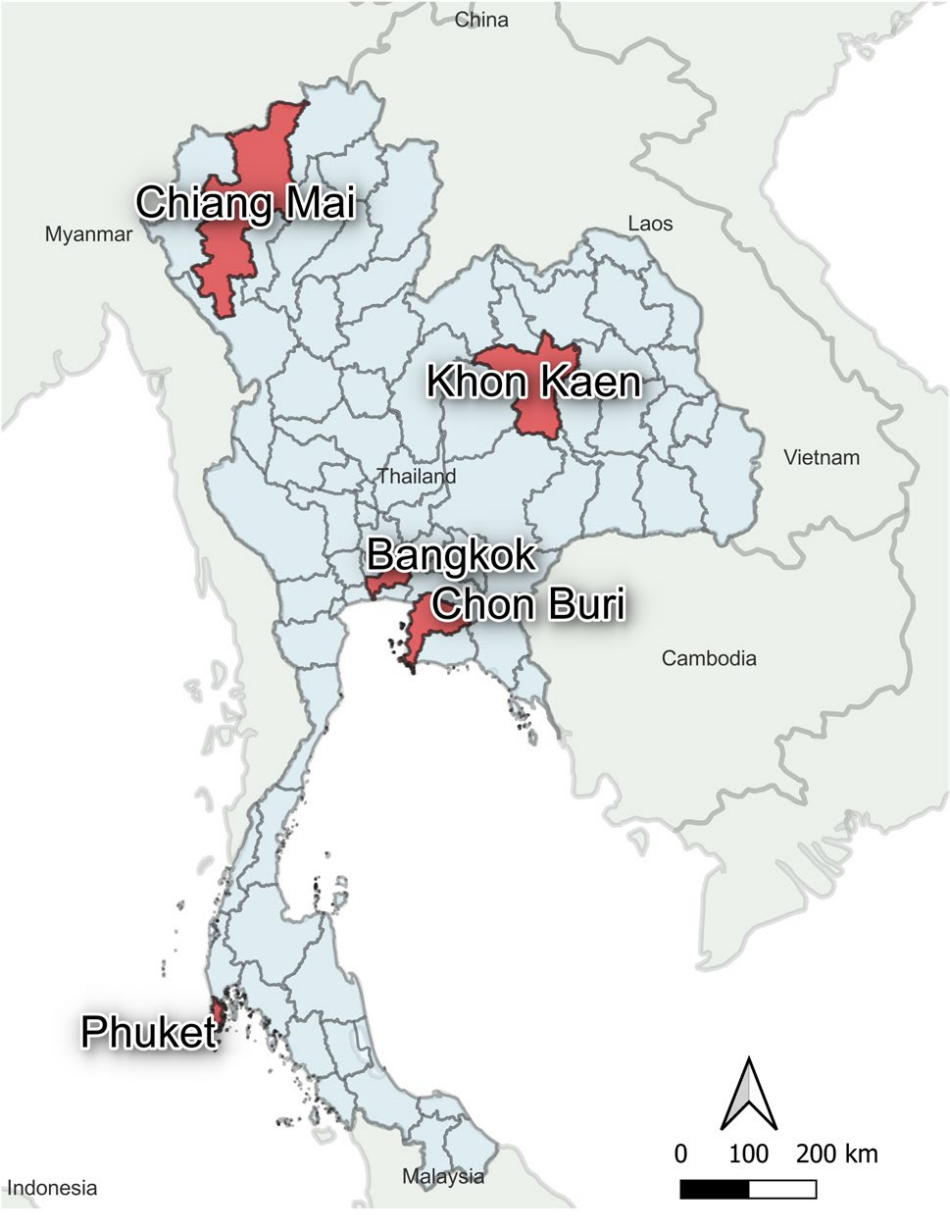
The distribution of blood centers involved in the study. 5 blood centers from 5 provinces in Thailand were enrolled in the present study (red colored regions in the map). The map was generated by geographical information system QGIS 3.28 software.

Donor blood samples were randomly selected from healthy volunteers with no past medical history of any significant infection or illness. All volunteers were confirmed to be healthy by physical examination. Blood samples were obtained from the National Blood Center in Bangkok (N = 218) and four regional blood centers located in Chiang Mai (N = 201), Khon Kaen (201), Chon Buri (N = 233), and Phuket province (N = 200) between March to September 2020 as shown in Fig. 1. We aimed to include similar numbers of volunteers from each regional blood bank. Participants were approached and asked to be a volunteer for the study when they arrived at the blood bank. All participants gave written informed consent, and the study was conducted according to the Helsinki Declaration and Good Clinical Practice guidelines. The study protocol was approved by the Research Ethics Committee of the National Blood Center, the Thai Red Cross (No. NBC 13/2020). Whole blood samples were collected into a serum collection tube (red topped tube) and sat in an undisturbed upright position for at least 15–30 min at room temperature to allow the blood to clot. The tube was centrifuged for 10 min at 3000 RPM. The liquid component (serum) was immediately transferred into a clean polypropylene tube and stored in aliquots at – 80 °C for further analysis. Demographic data on blood donors was obtained for all samples.

### Detection of anti-Leptospira IgG antibody

All ssoftware…ples were analyzed for anti-*Leptospira* IgG antibodies using commercially available enzyme immunoassay (SERION ELISA classic *Leptospira* IgG, catalog number ESR125G, Institut Virion/Serion GmbH, Warburg, Germany).

The kit is designed to detect IgG antibodies against all *Leptospira* species. The kit uses a native membrane extract from *Leptospira biflexa* with genus-specific antigens. It is able to detect human antibodies against *Leptospira* spp, including all pathogenic *Leptospira*. The kit was used in several seroprevalence studies^42–44^. The assays were performed following the manufacturer’s instructions.

Briefly, 10 μL of serum sample was diluted with 1,000 μL of dilution buffer and mixed thoroughly to ensure the solutions were homogenous. Then 100 μL of the diluted sample or ready-to-use controls were added to wells of the microtiter test strips and incubated at 37 °C for 60 min in a moist chamber. After incubation, wells were washed four times with 300 μL washing solution, and 100 μL of ready-to-use anti-human-IgG from goat (polyclonal), conjugated to alkaline phosphatase, was added to the wells and incubated at 37 °C for 30 min in the moist chamber. Following the second incubation, all wells were washed four times with 300 μL washing solution, and 100 μL of ready-to-use para-nitrophenylphosphate substrate was added, followed by incubation at 37 °C for 30 min in the moist chamber. Finally, 100 μL of 1.2 N sodium hydroxide-stopping solution was added to each well, and the microtest plate was gently shaken to mix. The optical density (OD) was read within 60 min at 405 nm against substrate blank. Qualitative analysis was evaluated for IgG antibodies. The cut-off value was calculated by multiplying the mean value of the measured standard-OD with the numerical data of the certificate of quality control. The range of the cutoff was in between 0.497 and 0.627. According to the kit’s insert, the assay used has a sensitivity of 94.7% and a specificity of > 99%. Positive and negative controls were included in each run.

### The microscopic agglutination test (MAT)

All samples were also tested by microscopic agglutination test (MAT) to detect prior *Leptospira* infection using a panel of 24 reference serovars including Australis, Autumnalis, Ballum, Bataviae, Canicola, Cellidoni, Cynopteri, Djasiman, Grippotyphosa, Hebdomadis, Icterohaemorrhagiae, Javanica, Louisaina, Manhao, Mini, Panama, Pomona, Pyrogenes, Ranarum, Sarmin, Sejroe, Shermani, Tarasovi, Semaranga. Sera were initially screened at 1:50 dilution and those showing 50% or more agglutination under a dark-field microscope were then serially diluted further to determine a titer endpoint. Samples with titers ≥ 1:50 were considered as past infection, while a titer of 1:400 or higher was used to define recent infection. This cut-off point has been used in other seroprevalence studies^37,45,46^.

### Statistical analysis

Statistical analysis was performed with IBM SPSS Statistics Version 22 (SPSS, Chicago, IL). Categorical data were expressed as numbers with percentages. Continuous variables are reported as mean and standard deviation in the case of a normal distribution and as a median and interquartile range in the case of non-normal distribution. We used a univariate logistic regression analysis to assess the relationship between gender, age, BMI, and living area with anti-*Leptospira* antibodies. If the p-value is lower than 0.05, the result is considered significant.

## Data Availability

The datasets generated during and/or analysed during the current study are available from the corresponding author on reasonable request.
